# Expedited Safety Reporting Through an Alert System for Clinical Trial Management at an Academic Medical Center: Retrospective Design Study

**DOI:** 10.2196/14379

**Published:** 2020-02-27

**Authors:** Yu Rang Park, HaYeong Koo, Young-Kwang Yoon, Sumi Park, Young-Suk Lim, Seunghee Baek, Hae Reong Kim, Tae Won Kim

**Affiliations:** 1 Department of Biomedical Systems Informatics Yonsei University College of Medicine Seoul Republic of Korea; 2 Clinical Research Center Asan Institute of Life Sciences Asan Medical Center Seoul Republic of Korea; 3 Clinical Trial Center Asan Medical Center Seoul Republic of Korea; 4 Department of Gastroenterology, Liver Center Asan Medical Center University of Ulsan College of Medicine Seoul Republic of Korea; 5 Department of Clinical Epidemiology and Biostatistics Asan Medical Center University of Ulsan College of Medicine Seoul Republic of Korea; 6 Department of Oncology Asan Medical Center University of Ulsan College of Medicine Seoul Republic of Korea

**Keywords:** clinical trial, adverse event, early detection, patient safety

## Abstract

**Background:**

Early detection or notification of adverse event (AE) occurrences during clinical trials is essential to ensure patient safety. Clinical trials take advantage of innovative strategies, clinical designs, and state-of-the-art technologies to evaluate efficacy and safety, however, early awareness of AE occurrences by investigators still needs to be systematically improved.

**Objective:**

This study aimed to build a system to promptly inform investigators when clinical trial participants make unscheduled visits to the emergency room or other departments within the hospital.

**Methods:**

We developed the Adverse Event Awareness System (AEAS), which promptly informs investigators and study coordinators of AE occurrences by automatically sending text messages when study participants make unscheduled visits to the emergency department or other clinics at our center. We established the AEAS in July 2015 in the clinical trial management system. We compared the AE reporting timeline data of 305 AE occurrences from 74 clinical trials between the preinitiative period (December 2014-June 2015) and the postinitiative period (July 2015-June 2016) in terms of three AE awareness performance indicators: onset to awareness, awareness to reporting, and onset to reporting.

**Results:**

A total of 305 initial AE reports from 74 clinical trials were included. All three AE awareness performance indicators were significantly lower in the postinitiative period. Specifically, the onset-to-reporting times were significantly shorter in the postinitiative period (median 1 day [IQR 0-1], mean rank 140.04 [SD 75.35]) than in the preinitiative period (median 1 day [IQR 0-4], mean rank 173.82 [SD 91.07], *P*≤.001). In the phase subgroup analysis, the awareness-to-reporting and onset-to-reporting indicators of phase 1 studies were significantly lower in the postinitiative than in the preinitiative period (preinitiative: median 1 day, mean rank of awareness to reporting 47.94, vs postinitiative: median 0 days, mean rank of awareness to reporting 35.75, *P*=.01; and preinitiative: median 1 day, mean rank of onset to reporting 47.4, vs postinitiative: median 1 day, mean rank of onset to reporting 35.99, *P*=.03). The risk-level subgroup analysis found that the onset-to-reporting time for low- and high-risk studies significantly decreased postinitiative (preinitiative: median 4 days, mean rank of low-risk studies 18.73, vs postinitiative: median 1 day, mean rank of low-risk studies 11.76, *P*=.02; and preinitiative: median 1 day, mean rank of high-risk studies 117.36, vs postinitiative: median 1 day, mean rank of high-risk studies 97.27, *P*=.01). In particular, onset to reporting was reduced more in the low-risk trial than in the high-risk trial (low-risk: median 4-0 days, vs high-risk: median 1-1 day).

**Conclusions:**

We demonstrated that a real-time automatic alert system can effectively improve safety reporting timelines. The improvements were prominent in phase 1 and in low- and high-risk clinical trials. These findings suggest that an information technology-driven automatic alert system effectively improves safety reporting timelines, which may enhance patient safety.

## Introduction

In recent trends of drug development, proof of concept is rapidly achieved at a low cost, allowing successful projects to be promptly positioned for late-stage development [1,2]; thus, it is critical that investigators be notified of the early signs of a drug’s efficacy and safety in real time during clinical trials. The solutions to the challenges of postmarketing evaluation of drug safety require highly collaborative interactions among the US Food and Drug Administration, industry, and other health authorities, as well as the development of registries for spontaneous reporting and epidemiological statistical methodology to detect and interpret adverse event (AE) signals [3-6]. Although clinical trials have rigorous procedures for reporting AEs, few studies have investigated how to detect and report them [7-9].

For reporting, the Consolidated Standards of Reporting Trials (CONSORT) Group generated recommendations regarding the appropriate reporting of AEs [8,9]. With advances in information technology (IT), the reporting of safety and other clinical trial data is moving from paper-based to electronic formats. Typically, electronic reporting portals developed by the clinical trial sponsors and contract research organizations are used as centralized electronic repositories for ongoing clinical trial information to reduce time and cost associated with recordkeeping.

To ensure the safety of study participants, it is crucial to promptly manage AEs and serious adverse events (SAEs), especially during high-risk or early-phase clinical trials. AE management consists of detection, processing, and reporting. AEs in clinical trials are usually detected during scheduled visits or when participants inform investigators of unscheduled visits to the emergency department or clinic. Unscheduled visits are sometimes detected by coordinators during the review of patients’ electronic health records. Delayed awareness of AEs among study personnel may jeopardize patient safety, so the prompt detection of unscheduled visits is important during clinical trials. While many health care practitioners understand the importance of AE awareness and reporting in the clinical trial field, they face practical hurdles in systematically managing AEs.

To build a systematic AE management process that addresses issues from occurrence to awareness and reporting, IT support with clinical trial management systems (CTMSs) and electronic medical records (EMRs) can be useful. In July 2015, we established an alert system called the *Adverse Event Awareness System* (AEAS), which was derived from CTMSs and EMRs [10,11]. The AEAS promptly informs investigators when clinical trial participants make unscheduled visits to the emergency room or other departments within the hospital. Such notifications were designed to improve the timelines of AE reporting to stakeholders, such as sponsors, institutional review boards (IRBs), or regulatory bodies. This study investigated the effectiveness of the AEAS by analyzing and comparing the relevant AE reporting timelines before and after the establishment of the AEAS.

## Methods

### Study Setting

This study was conducted at Asan Medical Center (AMC), a tertiary hospital in Seoul, South Korea. AMC is the largest medical center in Korea, with approximately 2700 inpatient beds and 10,000 outpatient visits per day. AMC has been fully accredited by the Association for the Accreditation of Human Research Protection Program since 2013; the IRB at AMC has received accreditation from the Forum for Ethical Review Committees in the Asian and Western Pacific Region since 2006.

In the process of clinical trials, AMC follows the US Food and Drug Administration regulations, as well as the Korean Good Clinical Practice and International Conference on Harmonization guidelines for AE reporting, which stipulate that all SAEs must be immediately reported to the sponsor except for those that the protocol or Investigator’s Brochure identifies as not requiring immediate reporting [12,13]. Sponsors are also required to report fatal and life-threatening *suspected unexpected serious reactions* (SUSARs) to regulatory agencies within 7 days after the sponsor’s initial receipt of the information, while other SUSARs may be reported within 15 days.

To manage clinical trials, we implemented a site-specific CTMS in December 2014 [10]. A CTMS is a system for managing clinical trials and research data. The requirements of various organizations related to clinical trials were developed for 14 months and implemented for 12 months based on the Java-based Spring Framework 4.0 (Pivotal Software). The CTMS was developed as an all-in-one system that links hospital information systems, the electronic IRB, enterprise resource planning, and biomaterial management systems in the respective academic medical center. The AEAS was implemented in July 2015 as a submodule of the CTMS interfaced with the EMR to facilitate early awareness of AE occurrences by investigators and study coordinators. The detailed operation process is as follows: if a patient makes an unscheduled visit to the hospital, the EMR confirms that the patient is in a clinical trial; if the patient participates in a specific clinical trial, the patient’s information is sent to the CTMS through the application programming interface; the CTMS then sends a text notification to the principal investigator or clinical research coordinator regarding the patient’s emergency room visit or hospitalization.

### Selection of Clinical Trials

From December 2014 to June 2016, 196 clinical trials were managed through the AMC CTMS. Among these trials, 93 had at least one AE report, with a total of 305 initial, 403 follow-up, and 179 finalized AE reports. In this study, we included 305 initial AE reports from 74 clinical trials to measure the improvement in AE awareness by the implementation of the AEAS. The follow-up or finalized reports were excluded from this study because they could not serve to verify the effectiveness of the AEAS in matters such as early recognition of AEs.

**Figure 1:**
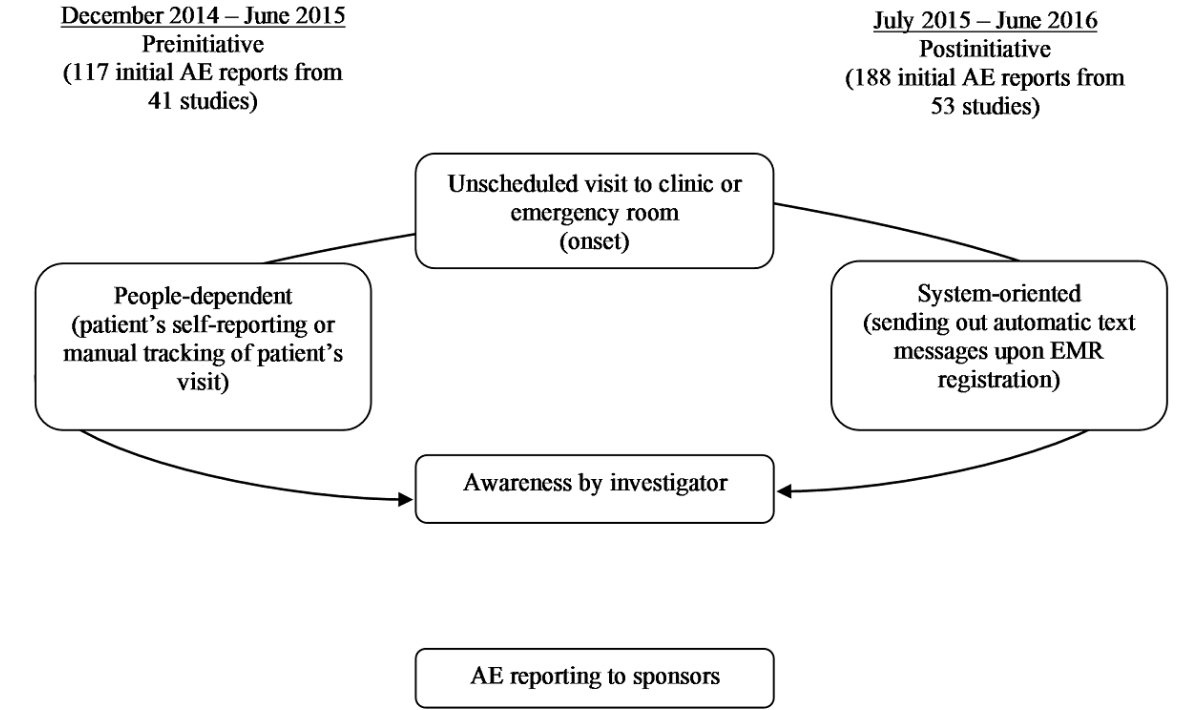
Initial adverse event (AE) reports before and after implementation of the Adverse Event Awareness System (AEAS). CTMS: clinical trial management system; EMR: electronic medical record.

A total of 117 initial AEs were processed in the CTMS prior to the implementation of the AEAS—December 2014 to June 2015—and 188 initial AEs were processed postimplementation—July 2015 to June 2016 (see Figure 1). Overall, patient AE monitoring processes were the same in the pre- and postinitiative periods, but the AE awareness method was different: people-dependent versus system-supported. Of the 74 clinical trials that were evaluated, 41 were carried out during the preinitiative period and 53 during the postinitiative period, with 20 trials overlapping the two periods.

The following data were collected: the dates of initial AE onset when investigators became aware of a given AE and when the AE report was submitted. The intervals between each step were calculated, with AE onset defined as the time when patients made an unscheduled visit to the emergency room or clinics.

### Statistical Analysis

General characteristics were compared using chi-square tests. The numbers of days between AE reporting phases (ie, onset, awareness, and reporting) were presented as medians with IQRs and mean ranks with SDs among the periods before and after AEAS implementation. The Shapiro-Wilk normality test showed the time intervals between AE onset, awareness, and reporting to have nonnormal distributions. We, therefore, used the Wilcoxon rank-sum test for comparing the pre- and postinitiative periods. We also presented the effect size of a pre- and postcomparison with a Cohen *d* test. According to US Food and Drug Administration guidelines, AEs are reported as being on business days [14-16]; likewise, we set the intervals using business days. We also performed subgroup analyses according to risk level and study phase. The risk level of each clinical trial was determined based on the classification by the ADApted MONitoring (ADAMON) project [17]. A higher risk level value means a subject has a higher than lower risk level. *P* values lower than .05 were deemed statistically significant. All analyses were performed in R, version 3.5.3 (The R Foundation).

### Ethics Statement

This study was approved by the IRB of AMC (IRB No. 2015-1368). The need for informed consent was waived by the ethics committee on the basis that this study utilized routinely collected medical data that were anonymously managed at all stages, including data cleaning and statistical analyses.

## Results

### Basic Characteristics

A total of 305 initial AE reports from 74 clinical trials were included in this study (see Table 1). Most initial AE reports subjected to analysis occurred in sponsor-initiated trials (286/305, 93.8%) and multisite trials (273/305, 89.5%). Initial AE reports were more common in phase 3 trials, followed by phase 1 and 2 trials; approximately 70% of initial AE reports were from risk-level 3 trials (210/305, 68.9%). Most of the basic characteristics of the clinical trials were not significantly different between the pre- and postinitiative periods. The frequency of global trials was higher in the postinitiative period.

**Table 1:** Basic characteristics of initial adverse event (AE) reports from 74 clinical trials.

Category		Total (N=305), n (%)	Preinitiative (N=117), n (%)	Postinitiative (N=188), n (%)	*P* value^a^
**Type of clinical trial**					.46
	Investigator-initiated trial	19 (6.2)	9 (7.6)	10 (5.3)	
	Sponsor-initiated trial	286 (93.8)	109 (92.4)	177 (94.7)	
**Number of sites involved**					.06
	Multisite	273 (89.5)	110 (93.2)	163 (87.2)	
	Single site	32 (10.5)	7 (5.9)	25 (13.4)	
**Phase**					.08
	1	78 (25.6)	24 (20.3)	54 (28.9)	
	2	56 (18.4)	26 (22.0)	30 (16.0)	
	3	117 (38.4)	33 (28.0)	84 (44.9)	
	4 and other	54 (17.7)	34 (28.8)	20 (10.7)	
**Scope of trial**					.009
	Domestic	48 (15.7)	27 (22.9)	21 (11.2)	
	Global	257 (84.3)	90 (76.3)	167 (89.3)	
**Risk level**					.32
	1	28 (9.2)	11 (9.3)	17 (9.1)	
	2	67 (22.0)	20 (16.9)	47 (25.1)	
	3	210 (68.9)	86 (72.9)	124 (66.3)	
Number of clinical trials		74 (24.3)	41 (34.7)	53 (28.3)	

^a^Chi-square test.

### Adverse Event Awareness Performance

**Figure 2:**
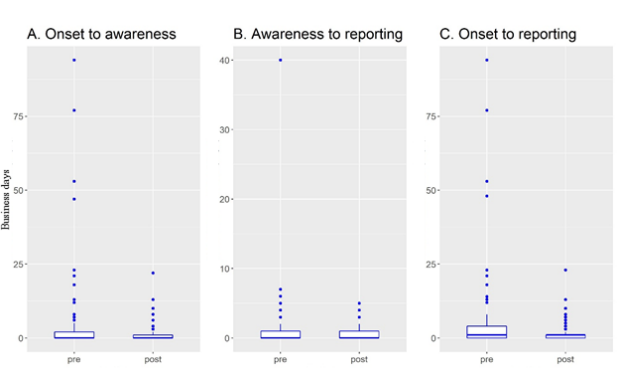
Date difference comparison between preinitiative (pre) and postinitiative (post) periods by adverse event (AE) awareness performance indicators.

All three AE awareness performance indicators were significantly lower in the postinitiative phase (see Table 2 and Figure 2). However, due to the skewed distribution (see Figure 2), median values were 0 days in both the pre- and postinitiative phases. Statistical testing found the distributions to be significantly lower, and examination of the mean rank demonstrated that reporting times were lower and more consistent overall in the postinitiative phase. In particular, the onset to reporting showed a statistically significant difference (preinitiative: median 1 day, mean rank 173.82, vs postinitiative: median 1 day, mean rank 140.04, *P*<.001). The onset-to-awareness and awareness-to-reporting time also showed significant reductions between the pre- and postinitiative periods (preinitiative: median 0 days, mean rank 164.15, vs postinitiative: median 0 days, mean rank 146.06, *P*=.04; and preinitiative: median 1 day, mean rank 173.82, vs postinitiative: median 1 day, mean rank 140.04, *P*=.02, respectively).

Figure 2 shows the date difference between pre- and postinitiatives by three AE awareness indicators. For all three AE awareness indicators, the phenomenon of AE records taking more than 25 business days preinitiative disappeared postinitiative.

**Table 2:** Adverse event (AE) awareness efficiency, preinitiative and postinitiative.

AE awareness performance indicator	Preinitiative (N=118 AEs)			Postinitiative (N=188 AEs)		*P* value^a^		Effect size^b^	
	Business days, median (IQR)	Mean rank (SD)	Business days, median (IQR)		Mean rank (SD)				
Onset to awareness	0 (0-2)	164.15 (83.41)	0 (0-1)		146.06 (68.67)		.04		0.117
Awareness to reporting	0 (0-1)	165.45 (79.58)	0 (0-1)		145.25 (67.36)		.02		0.135
Onset to reporting	1 (0-4)	173.82 (91.07)	1 (0-1)		140.04 (75.35)		<.001		0.197

^a^Wilcoxon rank-sum test.

^b^Effect sizes—0.1 (small effect), 0.3 (moderate effect), and 0.5 and above (large effect)—were calculated by dividing the absolute standardized test statistic *z* by the square root of the number of pairs.

### Adverse Event Awareness Performance by Phase and Risk

The phase subgroup analysis showed that all AE awareness indicators were reduced from the preinitiative period (see Table 3). The awareness-to-reporting and onset-to-reporting indicators in phase 1 studies showed statistically significant differences in median values and mean rank pre- and postinitiative (preinitiative: median 1 day, mean rank 47.94, vs postinitiative: median 0 days, mean rank 35.75, *P*=.01; and preinitiative: median 1 day, mean rank 47.4, vs postinitiative: median 1 day, mean rank 35.99, *P*=.03, respectively). In phase 4, there was no statistically significant difference in the onset-to-reporting timeline, but the SD of postinitiative ranks was significantly smaller than that of preinitiative ranks (preinitiative: rank SD 16.41 vs postinitiative: rank SD 11.89). Phase 3 and 4 studies showed greater differences than did phase 1 and 2 studies in the pre- and postinitiative SD values of all AE awareness performance indicators.

In the risk-level subgroup analysis, decreases of all AE report timelines were observed between pre- and postinitiative periods (see Table 4). Onset to awareness and onset to reporting of low-risk trials (level 1) showed significant reductions in the median and mean rank (preinitiative: median 4 days, mean rank 18.91, vs postinitiative: median 0 days, mean rank 11.65, *P*=.02; and preinitiative: median 4 days, mean rank 18.73, vs postinitiative: median 1 day, mean rank 11.76, *P*=.02, respectively). In high-risk trials (level 3), the distributions of awareness to reporting and onset to reporting were significantly different pre- and postinitiative (preinitiative: median 0 days, mean rank 115.94, vs postinitiative: median 0 days, mean rank 98.26, *P*=.02; and preinitiative: median 1 day, mean rank 117.36, vs postinitiative: median 1 day, mean rank 97.27, *P*=.01, respectively).

**Table 3:** Adverse event (AE) awareness efficiency, preinitiative and postinitiative, by study phase.

AE awareness performance indicator by phase		Preinitiative			Postinitiative				*P* value^a^		Effect size^b^			
		Business days, median (IQR)	Mean rank (SD)	Number of records		Business days, median (IQR)	Mean rank (SD)	Number of records						
**Phase 1 (n=22 studies, n=78 AEs)**														
	Onset to awareness	0 (0-1)	42.04 (19.86)	24		0 (0-0.75)	38.37 (17.04)	54		.41		0.09		
	Awareness to reporting	1 (0-1)	47.94 (20.61)			0 (0-1)	35.75 (18.55)			.01		0.28		
	Onset to reporting	1 (0.75-2.50)	47.4 (21.56)			1 (0-1)	35.99 (20.24)			.03		0.25		
**Phase 2 (n=14 studies, n=56 AEs)**														
	Onset to awareness	0 (0-1.75)	28.67 (16.42)	26		0.5 (0-1)	28.35 (13.65)	30		.94		0.01		
	Awareness to reporting	1 (0-1.75)	31.19 (15.85)			0 (0-1)	26.17 (13.78)			.21		0.17		
	Onset to reporting	1.5 (0.25-4.75)	31.98 (17.07)			1 (0-2)	25.48 (14.38)			.13		0.20		
**Phase 3 (n=29 studies, n=117 AEs)**														
	Onset to awareness	0 (0-1)	60.98 (31.18)	33		0 (0-1)	58.22 (26.28)	84		.63		0.04		
	Awareness to reporting	0 (0-1)	61.12 (26.84)			0 (0-0)	58.17 (24.54)			.57		0.05		
	Onset to reporting	0 (0-1)	61.71 (34.64)			0 (0-1)	57.93 (29.55)			.55		0.05		
**Phase 4 and other (n=9 studies, n=54 AEs)**														
	Onset to awareness	0.5 (0-3.75)	29.68 (15.20)	34		0 (0-1)	23.80 (12.03)	20		.15		0.20		
	Awareness to reporting	0 (0-0)	27.72 (12.41)			0 (0-0.25)	27.13 (10.88)			.87		0.02		
	Onset to reporting	1 (0-6.75)	29.93 (16.41)			1 (0-1)	23.38 (11.89)			.13		0.21		

^a^Wilcoxon rank-sum test.

^b^Effect sizes—0.1 (small effect), 0.3 (moderate effect), and 0.5 and above (large effect)—were calculated by dividing the absolute standardized test statistic *z* by the square root of the number of pairs.

**Table 4:** Adverse event (AE) awareness efficiency in preinitiative and postinitiative periods according to risk level.

AE awareness performance indicator by risk level		Preinitiative			Postinitiative			*P* value^a^	Effect size^b^
		Business days, median (IQR)	Mean rank (SD)	Number of records	Business days, median (IQR)	Mean rank (SD)	Number of records		
**Level 1 (n=5 studies, n=28 AEs)**									
	Onset to awareness	4 (0.5-22.0)	18.91 (8.09)	11	0 (0-1)	11.65 (5.99)	17	.02	0.46
	Awareness to reporting	0 (0-0)	14.23 (6.10)		0 (0-0)	14.68 (5.90)		.87	0.03
	Onset to reporting	4 (1-23)	18.73 (8.35)		1 (0-1)	11.76 (6.30)		.02	0.43
**Level 2 (n=17 studies, n=67 AEs)**									
	Onset to awareness	0.5 (0-6.25)	39.75 (20.31)	20	0 (0-1)	31.55 (15.27)	47	.08	0.22
	Awareness to reporting	0 (0-0)	33.73 (12.80)		0 (0-0)	34.12 (12.54)		.92	0.01
	Onset to reporting	0.5 (0-6.25)	37.65 (21.93)		1 (0-1)	32.45 (16.00)		.28	0.13
**Level 3 (n=52 studies, n=210 AEs)**									
	Onset to awareness	0 (0-1)	108.20 (53.50)	86	0 (0-1)	103.63 (47.69)	124	.52	0.05
	Awareness to reporting	0 (0-1)	115.94 (56.72)		0 (0-1)	98.26 (48.93)		.02	0.16
	Onset to reporting	1 (0-3)	117.36 (61.19)		1 (0-1)	97.27 (53.62)		.01	0.17

^a^Wilcoxon rank-sum test.

^b^Effect sizes—0.1 (small effect), 0.3 (moderate effect), and 0.5 and above (large effect)—were calculated by dividing the absolute standardized test statistic *z* by the square root of the number of pairs.

## Discussion

### Principal Findings

The most notable finding of this study is that the timeline from patients’ unscheduled visits (ie, onset), due to AE, to safety reporting was significantly reduced after the implementation of the AEAS. Also, the variability in the amount of time between patient visits and investigator awareness was lower after the implementation. This suggests that the AEAS notifications are effective for improving the speed with which investigators or clinical research coordinators are informed, thereby allowing them to take prompt action against AE occurrences.

There have been several approaches to enhance the efficiency, completeness, and consistency of safety reporting through the use of Web-based electronic safety reporting modules [18,19]. However, such approaches do not detect patients’ unscheduled visits at the site level. To our knowledge, this is the first study to evaluate the effectiveness of the implementation of an IT-driven AE awareness system for clinical trials at the site level. Early safety signal awareness with the alert system may contribute to better patient protection after the occurrence of AEs. A recent study found that many SAE results registered in ClinicalTrials.gov were yet to be published or omitted from publications [20]. Due to the imbalance of information on AE reporting, there is a high demand for more comprehensive approaches to ensure the safety of clinical trial participants [20-22]. There are only a few reports that have evaluated the timeline between AE onset and the initial reporting of the AE at the site level. One study reported that the mean duration from onset to reporting was 25 days for nonserious AEs and 11 days for SAEs [18]. However, to the best of our knowledge, there are no reports on other systems designed to improve the detection of AEs during clinical trials. Caution is necessary when comparing our findings with those of other studies because of the differences in protocols, safety reporting systems, and sites. However, our improved safety reporting metrics—from unscheduled visit to reporting—which were less than 2 days in the postinitiative period, deserve highlighting.

Another interesting finding is that the improvement in turnaround times after AEAS implementation was more prominent in phase 1 and in low- and high-risk clinical trials. The primary goal of a phase 1 study is to identify safety profiles and determine the dose-limiting toxicities of a new drug. This might be due to the increased attention being paid to participants in phase 1; therefore, the improvement in the reporting timeline was not as noticeable in trials of other phases as it was in phase 1 trials. We note that the safety reporting timeline is shorter postinitiative than preinitiative in all phases of clinical research.

### Limitations

The main limitation of our study is that our study results are based on a retrospective analysis of clinical trials carried out in a single academic medical center. Also, this system could not detect patient visits to other hospitals, which would require patient self-reporting; the rate of patient self-reporting may be improved with education, telephone monitoring, and questioning during trial visits. Another limitation is that we only analyzed the timeline for reporting AEs. Nevertheless, detailed analyses of AEs in clinical trials were not accessible at this point, as such analysis is only possible when the data are reported to the regulatory agency and published. Due to the study’s retrospective nature, there is a trend of imbalances among several characteristics, such as phase of trial or number of sites.

### Conclusions

In this study, we demonstrated that the AEAS, a CTMS-driven real-time automatic alert system, can effectively improve safety reporting timelines. The AEAS resulted in overall reductions in AE awareness timelines in all clinical trials, especially in phase 1 trials and low- and high-risk studies. These findings suggest that IT-driven automatic alert systems are effective in improving safety reporting timelines, which may ultimately enhance patient safety.

## Abbreviations

ADAMON: ADApted MONitoring
AE: adverse event
AEAS: Adverse Event Awareness System
AMC: Asan Medical Center
CONSORT: Consolidated Standards of Reporting Trials
CTMS: clinical trial management system
EMR: electronic medical record
IRB: institutional review board
IT: information technology
SAE: serious adverse event
SUSAR: suspected unexpected serious reaction
